# Association between mothers’ fish intake during pregnancy and infants’ sleep duration: a nationwide longitudinal study—The Japan Environment and Children’s Study (JECS)

**DOI:** 10.1007/s00394-021-02671-4

**Published:** 2021-09-09

**Authors:** Narumi Sugimori, Kei Hamazaki, Kenta Matsumura, Haruka Kasamatsu, Akiko Tsuchida, Hidekuni Inadera, The Japan Environment Children’s Study Group, Michihiro Kamijima, Shin Yamazaki, Yukihir Ohya, Reiko Kishi, Nobuo Yaegashi, Koichi Hashimoto, Chisato Mori, Shuichi Ito, Zentaro Yamagata, Hidekuni Inadera, Takeo Nakayama, Hiroyasu Iso, Masayuki Shima, Youichi Kurozawa, Narufumi Suganuma, Koichi Kusuhara, Takahiko Katoh

**Affiliations:** 1 Department of Public Health, Faculty of Medicine, University of Toyama, 2630 Sugitani, Toyama City, Toyama 930-0194 Japan; 2 Toyama Regional Center for JECS, University of Toyama, Toyama, Japan

**Keywords:** Pregnancy, Fish, n-3 polyunsaturated fatty acids, Infant, Sleep duration

## Abstract

**Purpose:**

N-3 polyunsaturated fatty acids (n-3 PUFAs), which are an important nutrient for humans, are particularly essential to the growth and development of the central nervous system (CNS) in fetuses and infants. Consequently, sufficient n-3 PUFA intake by mothers during pregnancy is considered to contribute to CNS development in their infants. CNS development is known to be associated with sleep, but no large epidemiological studies have yet confirmed that n-3 PUFA intake during pregnancy is associated with infants’ sleep.

**Methods:**

After exclusion and multiple imputation from a dataset comprising 104 065 records from the Japan Environment and Children’s Study (JECS), we examined 87 337 mother–child pairs for the association between mothers’ fish and n-3 PUFA intakes and risk of their infants sleeping less than 11 h at 1 year of age.

**Results:**

Multiple logistic regression analysis with the lowest quintile used as a reference revealed odds ratios for the second through fifth quintiles of 0.81 (95% confidence interval [95% CI] 0.76–0.87), 0.81 (95% CI 0.76–0.87), 0.78 (95% CI 0.72–0.84), and 0.82 (95% CI 0.76–0.88) for fish intake (*p* for trend < 0.001) and 0.90 (95% CI 0.84–0.97), 0.88 (95% CI 0.81–0.94), 0.88 (95% CI 0.82–0.95), and 0.93 (95% CI 0.86–0.998) for n-3 PUFA intake (*p* for trend = 0.04).

**Conclusions:**

Low fish intake during pregnancy may increase the risk of infants sleeping less than 11 h at 1 year of age. This relationship may have been mediated by maternal n-3 PUFA intake and infant neurodevelopment, but further evidence from interventional and other studies is needed to determine the appropriate level of fish intake during pregnancy.

**Trial registration:**

The Japan Environment and Children’s Study, https://upload.umin.ac.jp/cgi-open-bin/ctr_e/ctr_view.cgi?recptno=R000035091 (Registration no. UMIN000030786).

**Supplementary Information:**

The online version contains supplementary material available at 10.1007/s00394-021-02671-4.

## Introduction

N-3 polyunsaturated fatty acids (n-3 PUFAs) constitute a class of unsaturated fatty acids that is essential for maintaining human health. Among the well-known PUFAs, alpha-linolenic acid (ALA) is found in high concentrations in vegetable oil and eicosapentaenoic acid (EPA), docosapentaenoic acid (DPA), and docosahexaenoic acid (DHA) are abundant in seafood. N-3 PUFAs, particularly DHA, are found in high concentrations in the human central nervous system (CNS) as constituent fatty acids of phospholipids [1], and they play a crucial role in maintaining the structure and function of the CNS. However, the human body is not capable of de novo synthesis of ALA, so we must obtain it from foods [2]. Although the human body is capable of converting ALA to EPA, DPA, and DHA, the rates are very low [2]. Naturally, the same is true for fetuses, who must take in n-3 PUFAs via the placenta [3]. In the third trimester of pregnancy, as the weight of the fetal CNS increases dramatically, the demand for n-3 PUFAs is vastly increased [4]. It would appear then that maternal diet is critical for good development of the fetal CNS. In a recent birth cohort study of over 80 000 mothers and their children, we found that the mothers’ intake of fish and n-3 PUFAs during pregnancy was significantly associated with the level of their infants’ neurodevelopment at 6 months and 1 year after delivery [5].

Neurodevelopment is regarded as being closely tied to sleep until 1 year of age [6]. So, how might mothers’ intake of n-3 PUFAs during pregnancy correlate with their infants’ sleep? Cheruku et al. studied how the DHA content of plasma phospholipids in 17 mothers in the immediate postpartum period correlated with the sleep pattern of their newborns [7]. They found that the newborns of mothers with high plasma DHA had a better sleep pattern and suggested that this finding reflected greater CNS maturity [7]. Judge et al. conducted a placebo-controlled double-blind study with 48 women and found that the newborns of mothers who took supplements containing DHA in the third trimester had more mature sleep/wake states than the infants of mothers who took the placebo. Infant sleep/wake states were measured on postnatal days 1 (D1) and 2 (D2), and it was found that there were significantly fewer arousals in the DHA intervention group compared with the placebo group on D1 (*P* = 0.006) and D2 (*P* = 0.011), as well as significantly fewer arousals in active sleep in the DHA intervention group compared with the placebo group on D1 (*P* = 0.012) [8]. A double-blind controlled study involving 395 children aged 7 to 9 years showed that a higher DHA concentration in total lipids from whole blood was associated with sleep quality and that sleep duration improved in those children who took supplements containing DHA [9]. The conclusion that can be drawn from all of these studies is that sufficient intake of either fish or n-3 PUFAs might be associated with either sleep quality or long sleep duration, and that this intake might be beneficial for the development of the CNS and maintaining its function. In addition, in a placebo-controlled double-blind study involving 49 male infants, Ogundipe et al. showed that the total cerebral volume including cerebrospinal fluid was significantly larger in infants born to pregnant women (*n* = 24) who took supplements containing DHA and arachidonic acid than in the placebo group (*n* = 25) (646.29 vs 579.91 mm^3^, respectively, *p* = 0.028), and they concluded that DHA intake is beneficial for CNS maturation given that the newborns of mothers who took supplements containing DHA had a larger brain volume than those of mothers who took the placebo [10]. Although many studies indicate that n-3 PUFA intake during pregnancy contributes to a good sleep pattern in infants, almost all studies to date have involved small populations. Research involving larger populations is therefore needed to validate their basic claims.

In this study, we examined data from the large population of over 100 000 participants involved in the ongoing Japan Environment and Children’s Study (JECS) in efforts to investigate the association between maternal intake of fish and n-3 PUFAs during pregnancy and the risk of infants sleeping less than 11 h at 1 year of age.

## Methods

### Study population

The JECS protocol has been described in detail elsewhere [11, 12]. Briefly, JECS, a nationwide government-funded birth cohort study, is evaluating the impact of certain environmental factors on child health and development. JECS participants were in the first trimester of pregnancy when enrolled from January 2011 to March 2014 in 15 regions across Japan [11, 12]. The eligibility criteria for participants (expectant mothers) were as follows: (1) resident in a study area at the time of recruitment and expected to reside continually in Japan for the foreseeable future, (2) expected delivery date between August 1, 2011 and mid-2014, and (3) able to participate in the study without difficulty (i.e., able to understand Japanese and to complete the self-administered questionnaire). Excluded were expectant mothers residing outside a study area even if they were visiting cooperating healthcare providers within a study area [11].

**Fig. 1:**
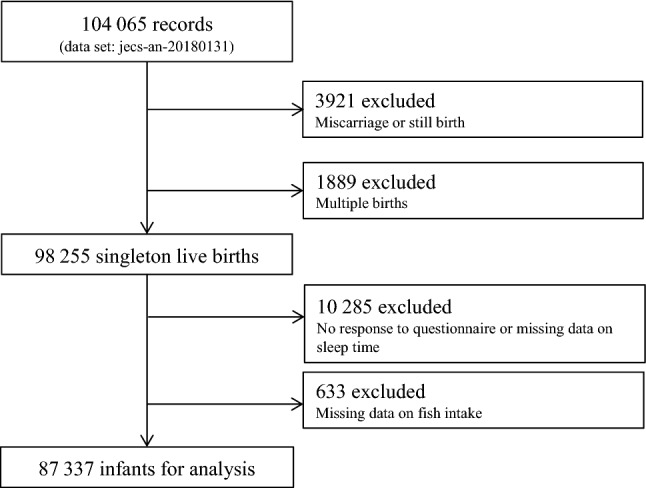
Flow diagram of the recruitment and exclusion process for the participants

The present study analyzed the jecs-an-20180131 dataset released in March 2018. The full dataset comprises 104 065 records obtained from self-reported questionnaires completed by the participants. We excluded 3921 and 1889 records because of miscarriages/still births and multiple births, respectively (Fig. 1), 10 285 records because of incomplete answers on the questionnaire, and 633 records for missing data on fish intake. This left 87,337 records for final analysis.

The JECS protocol was reviewed and approved by the Ministry of the Environment’s Institutional Review Board on Epidemiological Studies and the ethics committees of all participating institutions. All of the participants provided written informed consent.

### Measurements of fish and PUFA intake

In the JECS, dietary consumption of fish and total n-3 PUFAs was determined using the Food Frequency Questionnaire (FFQ), which is a semi-quantitative instrument that has been validated for use in large-scale Japanese epidemiologic studies [13]. Briefly, the FFQ was administered in mid-late pregnancy and participants were asked to respond about their dietary intake in the period between learning of their pregnancy and their second/third trimester. Of the 171 food and beverage items asked about in the FFQ, 21 concern fish or shellfish consumption. Participants answered how often they consumed each food type during the mid-late pregnancy (covering dietary intake after they learned of their pregnancy). The standard portion size for each food type was categorized as small (50% smaller than standard), medium (same as standard), or large (50% larger than standard). The nine frequency categories for each item were less than 1 time/month, 1–3 times/month, 1–2 times/week, 3–4 times/week, 5–6 times/week, every day, 2–3 times/day, 4–6 times/day, and ≥ 7 times/day. Daily intake of fish (g/day) was calculated as the frequency of consumption multiplied by the standard portion size for each fish item. The fatty acid composition table of Japanese foods [14] was used to calculate the daily intake of total n-3 PUFAs (the JECS dataset does not contain data for the individual subtypes of fatty acids) [14]. We performed log-transformation of fish and n-3 PUFA intakes and calculated the energy-adjusted intake using the residual model [15]. Because there were 4740 participants whose fish intake was 0 g/day, we replaced this value with 0.03 g/day, which is one-tenth of the lowest fish intake (0.3 g/day) of all participants (excluding 0 g/day). We did the same for 246 participants whose n-3 PUFA intake was 0 g/day, replacing it with 0.001 g/day, which is one-tenth of the lowest n-3 PUFA intake of all participants (excluding 0 g/day).

To measure the duration of infants’ sleep at 1 year of age, mothers were asked on the questionnaire to indicate when their infants slept on the previous day, by drawing lines through boxes indicating 30-min intervals from 12:00 to 12:00 am the next day. We chose 11 h as the cut-off for appropriate sleep duration in this study based on the recommendation by the United States National Sleep Foundation that 1-year-old infants sleep for 11–14 h in a 24-h period [15].

Covariates were adjusted for mother’s age, previous deliveries, body mass index at 1 month after delivery, highest educational level, annual household income, marital status at 6 months after delivery, alcohol intake at 1 month after delivery, smoking status at 1 month after delivery, employment status at 1 year after delivery, infant sex, infant attendance at nursery at age 1 year, where the infant slept at night, birth weight, gestational period, presence of congenital anomaly, date (month) of birth, the location where they were born, and presence of infant’s atopic dermatitis.

### Statistical analysis

Unless otherwise stated, data are expressed as the mean ± standard deviation or median. To estimate the risk of infants sleeping less than 11 h, we categorized participants according to quintile for fish or n-3 PUFA intake. Odds ratios (ORs) and 95% confidence intervals (CIs) were calculated using logistic regression analysis, with the lowest quintile used as a reference. Adjusted ORs were calculated using the covariates mentioned in the previous section (Measurements of fish and PUFA intake), and crude ORs were calculated without using these covariates. In tests for trend, categorical numbers were assigned to the quintile distributions for intake of each fish or n-3 PUFA item and were evaluated as continuous variables. In an exploratory analysis, we divided the participants into two groups according to the timing of FFQ administration (mid- vs late pregnancy) to examine whether there is a difference in effects between the two groups. We performed multiple imputations for any missing covariate values using chained equations to obtain five imputed datasets [16]. We included auxiliary variables related to the covariates to preserve the assumption of missing at random. Statistical significance was set at a two-sided *p* value of < 0.05. Analyses were performed with SAS version 9.4 (SAS Institute Inc., Cary, NC).

## Results

Table 1 shows the results for each covariate according to quintile for fish intake. Increased fish intake was associated with higher household income, higher educational level, and less smoking history. Table S1 shows the results for each covariate according to quintile for n-3 PUFA intake, with similar trends to those for fish intake found.

**Table 1:** Characteristics according to quintile for fish intake during pregnancy (*n* = 87,337)

	Quintile of fish intake (median intake, g/day)									
	Q1 (4.7)		Q2 (16.1)		Q3 (25.9)		Q4 (37.7)		Q5 (60.2)	
	*n* = 17,467		*n* = 17,468		*n* = 17,467		*n* = 17,468		*n* = 17,467	
Maternal age, years, mean ± SD	30.6	± 5.1	31.3	± 4.9	31.5	± 4.9	31.7	± 4.8	31.6	± 5.0
BMI at 1 month after delivery, *n* (%)										
< 18.5	758	(4.6)	792	(4.7)	811	(4.8)	861	(5.1)	1002	(6.0)
18.5– < 25	13,073	(78.8)	13,342	(79.5)	13,468	(80.2)	13,406	(79.8)	13,209	(79.0)
≥ 25	2759	(16.6)	2643	(15.8)	2525	(15.0)	2533	(15.1)	2515	(15.0)
Previous deliveries, *n* (%)										
Nullipara	7883	(46.2)	6966	(40.9)	6728	(39.4)	6621	(38.8)	6813	(40.1)
Multipara	9176	(53.8)	10,081	(59.1)	10,362	(60.6)	10,453	(61.2)	10,197	(60.0)
Annual household income (JPY), *n* (%)										
< 4 million	7296	(45.8)	6561	(40.1)	6307	(38.5)	5974	(36.3)	5983	(36.7)
4–6 million	4997	(31.3)	5474	(33.5)	5569	(34.0)	5591	(34.0)	5600	(34.3)
> 6 million	3650	(22.9)	4317	(26.4)	4528	(27.6)	4898	(29.8)	4737	(29.0)
Highest educational level, *n* (%)										
Junior high school or high school	7108	(41.2)	6055	(34.8)	5736	(32.9)	5464	(31.4)	5978	(34.4)
Technical junior college, technical/vocational college or associate degree	7140	(41.4)	7559	(43.4)	7579	(43.5)	7538	(43.3)	7241	(41.6)
Bachelor’s degree, postgraduate degree	3010	(17.4)	3802	(21.8)	4103	(23.6)	4415	(25.4)	4174	(24.0)
Marital status, *n* (%)										
Married (including common law marriage)	16,620	(97.5)	16,838	(98.4)	16,903	(98.6)	16,907	(98.6)	16,823	(98.3)
Divorced or widowed	170	(1.0)	132	(0.8)	114	(0.7)	119	(0.7)	137	(0.8)
Other	250	(1.5)	136	(0.8)	128	(0.8)	125	(0.7)	156	(0.9)
Smoking status at 1 month after delivery, *n* (%)										
Never	9342	(54.0)	10239	(59.1)	10621	(61.2)	10792	(62.2)	10563	(61.0)
Smoked previously but quit before learning of pregnancy	4086	(23.6)	4080	(23.5)	3893	(22.4)	3956	(22.8)	3934	(22.7)
Smoked previously but quit after learning of pregnancy	2987	(17.3)	2403	(13.9)	2253	(13.0)	2091	(12.1)	2220	(12.8)
Currently smoking	889	(5.1)	614	(3.5)	594	(3.4)	514	(3.0)	609	(3.5)
Alcohol intake at 1 month after delivery, *n* (%)										
Never	15,840	(91.5)	15,933	(91.8)	15,990	(92.0)	15,905	(91.6)	15,827	(91.2)
Ex-drinker	766	(4.4)	725	(4.2)	758	(4.4)	793	(4.6)	782	(4.5)
1–3 times/month	485	(2.8)	475	(2.7)	441	(2.5)	461	(2.7)	503	(2.9)
≥ 1 time/week	220	(1.3)	224	(1.3)	197	(1.1)	203	(1.2)	236	(1.4)
Employed at 1 year after delivery, *n* (%)	8507	(49.5)	8336	(48.4)	8313	(48.2)	8095	(46.9)	7959	(46.2)
Infant sex (boy), *n* (%)	8947	(51.2)	8963	(51.3)	8965	(51.3)	8985	(51.4)	8925	(51.1)
Birth weight, mean ± SD	3025	± 418	3034	± 409	3034	± 404	3033	± 407	3014	± 411
Gestational weeks, mean ± SD	39.3	± 1.5	39.3	± 1.5	39.3	± 1.5	39.3	± 1.5	39.2	± 1.5
Congenital anomaly, *n* (%)	393	(2.3)	402	(2.3)	374	(2.1)	379	(2.2)	420	(2.4)
Infant attendance at nursery at age 1 year, *n* (%)	5154	(29.6)	4689	(26.9)	4640	(26.7)	4491	(25.8)	4393	(25.2)
Location where infant sleeps at night, *n* (%)										
In parent’s bed	13,308	(76.3)	13,491	(77.4)	13,344	(76.5)	13,403	(76.9)	13,302	(76.3)
In baby bed in parents’ bedroom	3995	(22.9)	3838	(22.0)	3957	(22.7)	3928	(22.5)	4023	(23.1)
In baby bed in another room	110	(0.6)	88	(0.5)	113	(0.7)	89	(0.5)	98	(0.6)
Other	23	(0.1)	23	(0.1)	25	(0.1)	18	(0.1)	20	(0.1)
Presence of infant’s atopic dermatitis, *n* (%)	715	(4.1)	737	(4.2)	778	(4.5)	755	(4.3)	759	(4.4)

*BMI* body mass index, *SD* standard deviation

Table 2 shows the prevalence and odds ratios for risk of infants sleeping less than 11 h according to quintiles for intake of fish and n-3 PUFAs. The prevalence of infants sleeping less than 11 h according to quintile for maternal intake of fish and n-3 PUFAs during pregnancy was 11.2%, 9.3%, 9.3%, 8.9%, and 9.2% for fish intake and 10.4%, 9.5%, 9.2%, 9.2%, and 9.6% for n-3 PUFA intake. The odds ratios for the second through fifth quintiles compared with the lowest quintile were 0.81 (95% confidence interval [95% CI] 0.76–0.87), 0.81 (95% CI 0.76–0.87), 0.78 (95% CI 0.72–0.84), and 0.82 (95% CI 0.76–0.88) for fish intake (*p* for trend < 0.001) and 0.90 (95% CI 0.84–0.97), 0.88 (95% CI 0.81–0.94), 0.88 (95% CI 0.82–0.95), and 0.93 (95% CI 0.86–0.998) for n-3 PUFA intake (*p* for trend = 0.04).

**Table 2:** Odds Ratios (95% Confidence Intervals) for 1-year-old infants for risk of sleeping less than 11 h according to quintile for maternal intake of fish and n-3 PUFAS during pregnancy (*n* = 87 337)

	Quintile of each exposure					*p*-value for trend
	Q1	Q2	Q3	Q4	Q5	
Fish intake						
Median, g/day^a^	4.7	16.1	25.9	37.7	60.2	
Subtotal, *n*	17,467	17,468	17,467	17,468	17,467	
Cases, *n*	1959	1619	1619	1548	1613	
Prevalence, %	(11.2)	(9.3)	(9.3)	(8.9)	(9.2)	
Crude odds ratio	1.00 (Ref.)	**0.81 [0.75, 0.87]**	**0.81 [0.75, 0.87]**	**0.77 [0.72, 0.83]**	**0.81 [0.75, 0.86]**	** < 0.001**
Adjusted odds ratio^b^	1.00 (Ref.)	**0.81 [0.76, 0.87]**	**0.81 [0.76, 0.87]**	**0.78 [0.72, 0.84]**	**0.82 [0.76, 0.88]**	** < 0.001**
n-3 PUFA intake						
Median, g/day^a^	0.98	1.32	1.57	1.84	2.31	
Subtotal, *n*	17,467	17,468	17,467	17,467	17,468	
Cases, *n*	1823	1659	1601	1602	1673	
Prevalence, %	(10.4)	(9.5)	(9.2)	(9.2)	(9.6)	
Crude odds ratio	1.00 (Ref.)	**0.90 [0.84, 0.97]**	**0.87 [0.81, 0.93]**	**0.87 [0.81, 0.93]**	**0.91 [0.85, 0.98]**	**0.004**
Adjusted odds ratio^b^	1.00 (Ref.)	**0.90 [0.84, 0.97]**	**0.88 [0.81, 0.94]**	**0.88 [0.82, 0.95]**	**0.93 [0.86, 0.998]**	**0.04**

Values in bold indicate significance

*PUFA* polyunsaturated fatty acid

^a^Dietary intake between learning of pregnancy and second/third trimester

^b^Covariates were adjusted for mother's age, previous deliveries, body mass index at 1 month after delivery, highest educational level, annual household income, marital status at 6 months after delivery, alcohol intake at 1 month after delivery, smoking status at 1 month after delivery, employment status at 1 year after delivery, infant sex, infant attendance at nursery at age 1 year, where the infant slept at night, birth weight, gestational period, presence of congenital anomaly, date (month) of birth, location where infant was born, and presence of infant’s atopic dermatitis

Table S2 shows the results of the exploratory analysis. After dividing the participants into those who returned the FFQ in mid-pregnancy and those who returned it in late pregnancy, the results were essentially the same for fish intake. However, for n-3 PUFA intake, the association disappeared in some quintiles in late pregnancy.

## Discussion

In this study, we found that low fish intake during pregnancy may increase the risk of infants sleeping less than lower limit of the recommended sleep duration of 11 h at 1 year of age. This result is consistent with previous epidemiological studies showing that consuming a large amount of seafood, particularly n-3 PUFAs, during pregnancy leads to good neurodevelopment in newborns. Our study design has advantages over those of previous studies. For example, Cheruku et al. had a small sample size of only 17 women [7], and Hansen et al. noted that longer gestational period was a mediating factor [17]. In contrast, our study is a large, nationwide observational study, involving more than 87,000 expectant mothers, and we found very little difference in the mediating factor of gestational period between quintiles.

In addition, as shown in Table 2, the intake level with the lowest adjusted odds ratio was 37.7 g/day in the fourth quintile for fish intake (median) and 1.57–1.84 g/day in the third and fourth quintiles for n-3 PUFA intake (median). This finding suggests that sleep duration should improve the most around the intake levels in the third and fourth quintiles. We do not know why the data exhibit this non-linear response, but the fact that the pattern was stronger for n-3 PUFAs indicates that it may relate to balance with n-6 PUFAs. This is because prostaglandin D2 derived from the n-6 PUFA arachidonic acid plays an important role in the CNS for sleep induction [18]. Also, the adjusted odds ratio was slightly lower for fish intake than for n-3 PUFA intake. This could be because fish contains numerous other substances beneficial for sleep (e.g., selenium, vitamin D, and/or calcium [19]).

We focused on the association between mothers’ intake of fish and n-3 PUFAs during pregnancy and sleep duration in their 1-year-old infants in this study, but we previously found that changes in intestinal flora impact sleep duration in 1-year-olds [20]. N-3 PUFA intake may therefore have had some effect on the pregnant mothers’ intestinal flora [21] that then caused changes in their infants’ duration of sleep at 1 year of age. However, this is purely conjecture at this stage, and further research is warranted.

When the sub-analysis was conducted according to the timing of the FFQ (i.e., in mid-pregnancy or late pregnancy), the association was not particularly different for fish intake, but it was for n-3 PUFA intake, with the association disappearing for intake in some quintiles in late pregnancy. One possible explanation is that the demand for DHA increases the most in the third trimester [4], and this may have been related to the observed loss of association in late pregnancy. Selective biomagnification of DHA into cord blood is also known to occur [22], and if the degree of biomagnification increases with each stage of pregnancy, this may explain the loss of association as well. Lastly, a third possible reason could involve epigenetics. Early pregnancy is reported to be a particularly sensitive developmental period when insults such as stress and inflammation can promote epigenetic programming changes that increase the risk of disease [23]. Because n-3 PUFAs have an anti-inflammatory effect [24], the association found in the full analysis (Table 2) might be mostly derived from the association found in mid-pregnancy, which would reflect the n-3 PUFA intake in early pregnancy more than it would the n-3 PUFA intake in late pregnancy.

The main strengths of our study were the large sample size of over 80,000 and the fact that the sample can be considered representative of mothers and infants in Japan, given that the JECS covers a wide geographic range across 15 regions. One limitation of our study is that we evaluated fish and fatty acid intake data from the expectant mothers semiquantitatively using a dietary questionnaire. A second limitation is that we evaluated infant sleep duration by totaling the number of boxes of 30-min intervals that mothers drew lines through on the questionnaire. Both of these methods (dietary questionnaire and evaluation of sleep duration) generate error that is characteristic of self-administered questionnaires that rely on memory; therefore, recall bias was unavoidable. However, as has been discussed in the literature [13], the correlation between calculations from questionnaires and actual dietary intake can be considered statistically reliable. It is possible that our semiquantitative calculations may have systematically deviated from actual intake, and thus it may be necessary to carefully consider whether setting daily recommended intake based directly on semiquantitative calculations is appropriate. Lastly, in this observational study, unmeasured residual factors such as health consciousness might have confounded the results. Maternal health consciousness might influence both the mother’s dietary pattern during pregnancy and her care of the infant. Because we do not have data on maternal health consciousness, we accounted for socioeconomic status instead, using variables such as annual house income and maternal highest education.

The clinical implications of our findings are that daily intake of at least 16.1 g/day of fish (median of the second quintile) may reduce the risk of 1-year-old infants sleeping less than the lower limit of the recommended sleep duration (11–14 h), considering that there was little variation in the odds ratio between the second through fifth quintiles for fish intake (OR range, 0.78–0.82) and there were significant differences for each of these quintiles compared with the lowest quintile. Although there were also significant differences for the second through fifth quintiles for n-3 PUFA intake, individual fatty acid data (i.e., EPA, DPA, and DHA) were not available to us, and thus further research that includes blood level measurements is warranted.

In conclusion, the results of this study suggest that sufficient intake of fish or n-3 PUFAs by mothers during pregnancy may reduce the risk of their infants sleeping less than 11 h per day at 1 year of age. It appears that this relationship may have been mediated by maternal intake of n-3 PUFAs in fish and infant neurodevelopment, but further evidence acquired through interventional and other studies is needed to determine what level of fish intake is appropriate during pregnancy.

## Supplementary Information

Below is the link to the electronic supplementary material.

Supplementary file1 Table S1. Characteristics according to quintile for n-3 PUFA intake during pregnancy (n = 87,337) (DOCX 20 KB)

Supplementary file2 Table S2. Sub-analysis^a^ of adjusted odds ratios (95% Confidence Intervals) for 1-year-old infants for risk of sleeping less than 11 hours according to quintile for maternal intake of fish and n-3 PUFAs during pregnancy (DOCX 15 KB)
