# Supplementary material for: Association between mothers’ fish intake during pregnancy and infants’ sleep duration: a nationwide longitudinal study—The Japan Environment and Children’s Study (JECS)
Source: Eur J Nutr. 2021 Sep 9;61(2):679–86. doi: 10.1007/s00394-021-02671-4 (PMC8854241; doi:10.1007/s00394-021-02671-4)
Supplement: Supplementary file 1 — Supplementary file1 Table S1. Characteristics according to quintile for n-3 PUFA intake during pregnancy (n = 87,337) (DOCX 20 KB) [file 394_2021_2671_MOESM1_ESM.docx]

**Table S1.** Characteristics According to Quintile for n-3 PUFA Intake During Pregnancy (n = 87 337)

|  | Quintile for n-3 PUFA intake (median intake, g/day) | | | | | | | | | |
| --- | --- | --- | --- | --- | --- | --- | --- | --- | --- | --- |
|  | Q1 (0.98 g) | | Q2 (1.32 g) | | Q3 (1.57 g) | | Q4 (1.84 g) | | Q5 (2.31 g) | |
|  | n = 17467 | | n = 17 468 | | n = 17 467 | | n = 17 467 | | n = 17 468 | |
| **Maternal age, years, mean ± SD** | 30.9 | ±5.2 | 31.2 | ±4.9 | 31.4 | ±4.9 | 31.6 | ±4.9 | 31.5 | ±4.9 |
| **Pre-pregnancy BMI, n (%)** |  |  |  |  |  |  |  |  |  |  |
| <18.5 | 708 | (4.3) | 750 | (4.5) | 812 | (4.8) | 914 | (5.4) | 1,040 | (6.2) |
| 18.5-<25 | 12,963 | (78.2) | 13,281 | (79.2) | 13,477 | (80.3) | 13,484 | (80.3) | 13,293 | (79.3) |
| ≥25 | 2,909 | (17.6) | 2,747 | (16.4) | 2,503 | (14.9) | 2,394 | (14.3) | 2,422 | (14.5) |
| **Previous deliveries, n (%)** |  |  |  |  |  |  |  |  |  |  |
| Nullipara | 7,420 | (43.4) | 7,020 | (41.1) | 6,838 | (40.1) | 6,737 | (39.6) | 6,996 | (41.1) |
| Multipara | 9,668 | (56.6) | 10,049 | (58.9) | 10,216 | (59.9) | 10,297 | (60.5) | 10,039 | (58.9) |
| **Annual household income (JPY), n (%)** |  |  |  |  |  |  |  |  |  |  |
| <4 million | 6,790 | (42.4) | 6,527 | (39.9) | 6,266 | (38.3) | 6,185 | (37.7) | 6,353 | (39.0) |
| 4-6 million | 5,080 | (31.7) | 5,466 | (33.4) | 5,478 | (33.5) | 5,645 | (34.4) | 5,562 | (34.1) |
| >6 million | 4,147 | (25.9) | 4,377 | (26.7) | 4,626 | (28.3) | 4,586 | (27.9) | 4,394 | (26.9) |
| **Highest educational level, n (%)** |  |  |  |  |  |  |  |  |  |  |
| Junior high school or high school | 6,713 | (38.9) | 6,200 | (35.6) | 5,784 | (33.2) | 5,644 | (32.4) | 6,000 | (34.5) |
| Technical junior college, technical/vocational college or associate degree | 7,218 | (41.8) | 7,359 | (42.2) | 7,528 | (43.3) | 7,535 | (43.3) | 7,417 | (42.7) |
| Bachelor’s degree, postgraduate degree | 3,335 | (19.3) | 3,865 | (22.2) | 4,092 | (23.5) | 4,237 | (24.3) | 3,975 | (22.9) |
| **Marital status, n (%)** |  |  |  |  |  |  |  |  |  |  |
| Married (including common law marriage) | 16,642 | (97.6) | 16,825 | (98.3) | 16,878 | (98.6) | 16,907 | (98.5) | 16,839 | (98.4) |
| Divorced or widowed | 174 | (1.0) | 130 | (0.8) | 129 | (0.8) | 112 | (0.7) | 127 | (0.7) |
| Other | 229 | (1.3) | 156 | (0.9) | 114 | (0.7) | 144 | (0.8) | 152 | (0.9) |
| **Smoking status, n (%)** |  |  |  |  |  |  |  |  |  |  |
| Never | 9,802 | (56.6) | 10,318 | (59.5) | 10,517 | (60.6) | 10,590 | (61.1) | 10,330 | (59.6) |
| Smoked previously but quit before learning of pregnancy | 3,961 | (22.9) | 3,943 | (22.8) | 3,973 | (22.9) | 4,035 | (23.3) | 4,037 | (23.3) |
| Smoked previously but quit after learning of pregnancy | 2,706 | (15.6) | 2,413 | (13.9) | 2,302 | (13.3) | 2,181 | (12.6) | 2,352 | (13.6) |
| Currently smoking | 842 | (4.9) | 661 | (3.8) | 569 | (3.3) | 535 | (3.1) | 613 | (3.5) |
| **Alcohol intake, n (%)** |  |  |  |  |  |  |  |  |  |  |
| Never | 15,857 | (91.5) | 15,906 | (91.7) | 15,970 | (91.9) | 15,962 | (91.9) | 15,800 | (91.1) |
| Ex-drinker | 747 | (4.3) | 768 | (4.4) | 765 | (4.4) | 750 | (4.3) | 794 | (4.6) |
| 1-3 times/month | 496 | (2.9) | 456 | (2.6) | 455 | (2.6) | 445 | (2.6) | 513 | (3.0) |
| ≥ 1 time/week | 224 | (1.3) | 225 | (1.3) | 184 | (1.1) | 210 | (1.2) | 237 | (1.4) |
|  |  |  |  |  |  |  |  |  |  |  |
| **Employed at 1 year after delivery, n (%)** | 8,745 | (50.8) | 8,503 | (49.3) | 8,294 | (48.2) | 7,993 | (46.4) | 7,675 | (44.6) |
|  |  |  |  |  |  |  |  |  |  |  |
| **Infant sex (boy), n (%)** | 8,966 | (51.3) | 8,992 | (51.5) | 8,898 | (50.9) | 8,979 | (51.4) | 8,950 | (51.2) |
|  |  |  |  |  |  |  |  |  |  |  |
| **Birth weight, mean ± SD** | 3033 | ±414 | 3033 | ±411 | 3035 | ±404 | 3026 | ±411 | 3013 | ±408 |
|  |  |  |  |  |  |  |  |  |  |  |
| **Gestational weeks, mean ± SD** | 39.26 | ±1.5 | 39.28 | ±1.5 | 39.31 | ±1.5 | 39.25 | ±1.5 | 39.25 | ±1.5 |
|  |  |  |  |  |  |  |  |  |  |  |
| **Congenital anomaly, n (%)** | 411 | (2.4) | 374 | (2.1) | 358 | (2.1) | 420 | (2.4) | 405 | (2.3) |
|  |  |  |  |  |  |  |  |  |  |  |
| **Nursery attendance, n (%)** | 5,049 | (29.0) | 4,894 | (28.1) | 4,677 | (26.9) | 4,409 | (25.3) | 4,338 | (24.9) |
| **Location where infant sleeps at night, n (%)** |  |  |  |  |  |  |  |  |  |  |
| In parent's bed | 13,382 | (76.7) | 13,348 | (76.6) | 13,385 | (76.7) | 13,383 | (76.7) | 13,350 | (76.6) |
| In baby bed in parents' bedroom | 3,931 | (22.5) | 3,960 | (22.7) | 3,945 | (22.6) | 3,934 | (22.6) | 3,971 | (22.8) |
| In baby bed in another room | 103 | (0.6) | 95 | (0.5) | 104 | (0.6) | 112 | (0.6) | 84 | (0.5) |
| Other | 22 | (0.1) | 30 | (0.2) | 14 | (0.1) | 15 | (0.1) | 28 | (0.2) |
|  |  |  |  |  |  |  |  |  |  |  |
| **Presence of infant's atopic dermatitis, n (%)** | 718 | (4.1) | 745 | (4.3) | 748 | (4.3) | 781 | (4.5) | 752 | (4.3) |

Abbreviations: BMI, body mass index; SD, standard deviation.
