# Supplementary material for: Association between mothers’ fish intake during pregnancy and infants’ sleep duration: a nationwide longitudinal study—The Japan Environment and Children’s Study (JECS)
Source: Eur J Nutr. 2021 Sep 9;61(2):679–86. doi: 10.1007/s00394-021-02671-4 (PMC8854241; doi:10.1007/s00394-021-02671-4)
Supplement: Supplementary file 2 — Supplementary file2 Table S2. Sub-analysisa of adjusted odds ratios (95% Confidence Intervals) for 1-year-old infants for risk of sleeping less than 11 hours according to quintile for maternal intake of fish and n-3 PUFAs during pregnancy (DOCX 15 KB) [file 394_2021_2671_MOESM2_ESM.docx]

**Table S2.** Sub-analysis^a^ of Adjusted Odds Ratios (95% Confidence Intervals) for 1-Year-Old Infants for Risk of Sleeping Less Than 11 Hours According to Quintile for Maternal Intake of Fish and n-3 PUFAs During Pregnancy

|  |  | Quintile of each exposure | | | | |
| --- | --- | --- | --- | --- | --- | --- |
| Adjusted odds ratio ^b^ |  | Q1 | Q2 | Q3 | Q4 | Q5 |
|  |  |  |  |  |  |  |
| Fish intake | FFQ in mid-pregnancy (n=47,019) | 1.00 (Ref.) | **0.82 [0.74, 0.90]** | **0.82 [0.74, 0.90]** | **0.79 [0.71, 0.87]** | **0.81 [0.74, 0.90]** |
|  | FFQ in late pregnancy (n=40,318) | 1.00 (Ref.) | **0.81 [0.73, 0.90]** | **0.81 [0.73, 0.90]** | **0.76 [0.69, 0.85]** | **0.82 [0.74, 0.91]** |
|  |  |  |  |  |  |  |
| n-3 PUFA intake | FFQ in mid-pregnancy (n=47,019) | 1.00 (Ref.) | **0.87 [0.78, 0.96]** | **0.89 [0.81, 0.98]** | **0.84 [0.76, 0.92]** | **0.90 [0.81, 0.99]** |
|  | FFQ in late pregnancy (n=40,318) | 1.00 (Ref.) | 0.94 [0.84, 1.04] | **0.86 [0.77, 0.95]** | 0.93 [0.84, 1.04] | 0.97 [0.87, 1.08] |
|  |  |  |  |  |  |  |

^a^ Sub-analysis was conducted according to the timing of the FFQ (i.e., mid- vs late pregnancy)

^b^ Covariates were adjusted for mother's age, previous deliveries, body mass index at 1 month after delivery, highest educational level, annual household income, marital status at 6 months after delivery, alcohol intake at 1 month after delivery, smoking status at 1 month after delivery, employment status at 1 year after delivery, infant sex, infant attendance at nursery, where the infant slept at night, birth weight, gestational period, presence of congenital anomaly, date (month) of birth, location where infant was born, and presence of infant’s atopic dermatitis.

Abbreviation: PUFA, polyunsaturated fatty acid.

Values in bold are significant.
